# Psoriasis Flare Following Interleukin-17 (IL-17) Inhibition and Recent Streptococcal Infection: A Case Report Highlighting Management Complexity

**DOI:** 10.7759/cureus.104614

**Published:** 2026-03-03

**Authors:** Puspita Hassan, Michelle Gallagher

**Affiliations:** 1 Dermatology, Michigan State University College of Osteopathic Medicine, East Lansing, USA

**Keywords:** biologic il-17 inhibitor, guttate psoriasis, ixekizumab, paradoxical psoriasis, pde4 inhibitors, psoriasis treatment

## Abstract

Psoriasis is a chronic immune-mediated inflammatory skin disease that has a wide range of clinical manifestations. Biologic agents such as interleukin-17 (IL-17) inhibitors are increasingly used for the management of moderate-to-severe psoriasis due to their ability to achieve similar therapeutic outcomes with better safety profiles than conventional systemic treatments, such as methotrexate or cyclosporine. While typically safer, there is a rare possibility of these agents exacerbating psoriasis. Moreover, a psoriasis flare may occur from various causes, such as an adverse reaction to treatments or streptococcal infections. This case illustrates the complexity of managing psoriasis in patients who have contraindications to systemic treatments, are unresponsive to standard therapies, and may have potential complications from streptococcal infection. It highlights the need to consider multiple therapeutic approaches, including the use of phosphodiesterase 4 (PDE4) inhibitors. We discuss the case of a 37-year-old female with worsening of psoriasis following a streptococcal infection and treatment with ixekizumab.

## Introduction

Psoriasis is a chronic skin disease that is triggered by a combination of genetics, environmental factors, and immune dysregulation. The hallmark of psoriasis is hyperproliferation of keratinocytes and infiltration of immune cells, which contributes to the sustained inflammatory state. This clinically manifests as well-demarcated red plaques with micaceous scale in estimated 90% of cases, phenotypically characterized as plaque psoriasis [1]. The next common phenotype is guttate psoriasis, which may occur independently or as a flare-up in the presence of pre-existing plaque psoriasis. Guttate psoriasis is more common in children and young adults and is strongly associated with the infectious agent β-hemolytic streptococcus [1]. It appears as small, red, teardrop-shaped papules commonly on the trunk and extremities [1,2].

For moderate-to-severe psoriasis, systemic treatment is indicated, often combined with topical and phototherapeutic agents. While traditional systemic treatments such as methotrexate and cyclosporin can be used to manage psoriasis, major limiting factors include poor tolerability, greater risk of organ toxicity, and several black box warnings [3,4]. Biologic agents are a newer and safer systemic option, with an aim to reduce the levels of specific pro-inflammatory cytokines often found in abnormally high levels in psoriasis. Among the injectable biologics, interleukin-17 (IL-17) inhibitors (ixekizumab, brodalumab) and interleukin-23 (IL-23) inhibitors (risankizumab, guselkumab) have the greatest short-term and long-term Psoriasis Area and Severity Index (PASI) 90 response rates [5,6].

A meta-analysis examining 28,424 patients across multiple studies treated with IL-17 inhibitors for psoriasis found that the three most common adverse events were infections, nasopharyngitis, and injection site reactions [7]. Paradoxical psoriasis was rare, reported in 42 out of 1,207 patients [7]. Further, a literature review examining 30 cases of IL-17 inhibitor-induced paradoxical psoriasis revealed that a previous history of psoriasis was a risk factor, with no standard method of follow-up due to variability among patients [8]. In this report, we present the case of a 37-year-old female experiencing a psoriasis flare from an unclear origin, potentially triggered by a streptococcal infection or paradoxical reaction from the IL-17 inhibitor, ixekizumab.

## Case presentation

**Figure 1:**
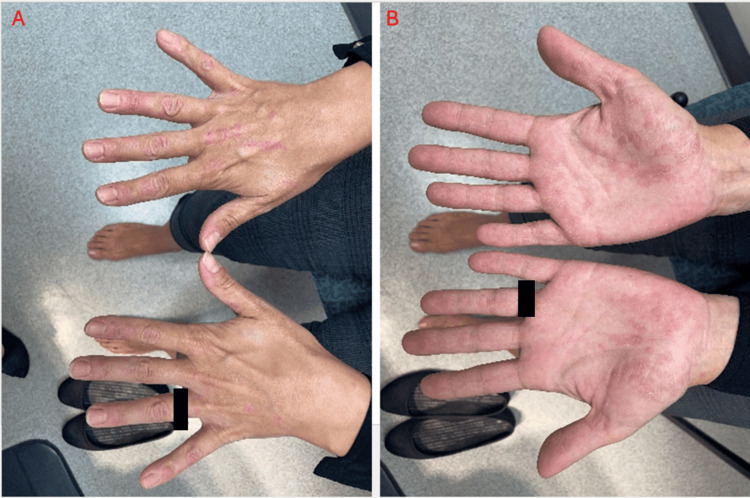
(A, B) Four months after starting ixekizumab, the patient developed a guttate flare-up with many new lesions, especially on the hands and feet.

**Figure 2:**
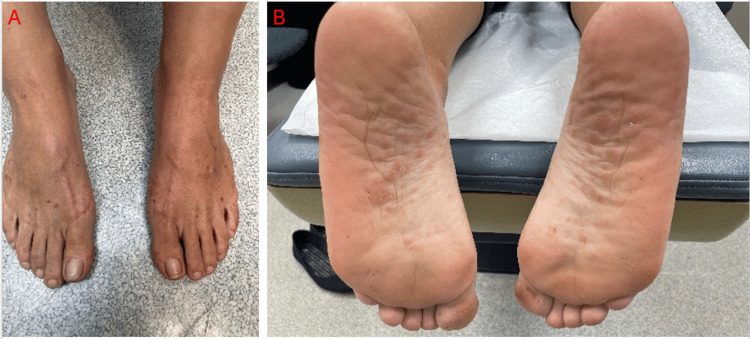
(A, B) Increased presence of scaly red papules in a guttate pattern in the dorsal and planter surfaces of the feet.

A 37-year-old female with a history of plaque psoriasis for 12 years experienced an inadequate response to topical and intralesional corticosteroids. Ixekizumab therapy was initiated in March 2023 with a 160 mg subcutaneous loading dose. Despite initial improvement, during a four-month follow-up, the patient exhibited worsening of psoriasis, featuring psoriasiform plaques with micaceous scale. There was also an eruption of multiple small guttate papules, which began palmoplantar and had spread to the left medial eyebrow, lower back, buttocks, upper and lower extremities (Figures 1, 2).

Prior to initiating ixekizumab therapy, the patient tried betamethasone diproprionate 0.05% gel, clobetasol 0.05% ointment, triamcinolone acetonide 0.1% ointment, and intralesional triamcinolone acetonide injections of 2.5 mg/mL to manage pruritus, but it continued to be constant in quality. Past medical history includes asthma, eczema, gestational diabetes mellitus, and gastric sleeve surgery. She is also a chronic smoker, with a 15-year history of smoking. She had clinical symptoms of joint pain and contraindications to systemic treatment with methotrexate and cyclosporine. The QuantiFERON-TB Gold test was negative. The patient was seen at an urgent care and treated with intramuscular ceftriaxone for a streptococcal infection on July 7, 2023, a week before the spots began on July 14. Group A streptococcus polymerase chain reaction (PCR) from a throat swab test was negative on July 25; however, an anti-streptolysin O (ASO) titer was not obtained. Therefore, a post-streptococcal worsening of psoriasis should be considered in the differential diagnosis.

After the presumed paradoxical reaction, the patient discontinued ixekizumab. She was trialed on deucravacitinib 6 mg once daily and narrowband ultraviolet B therapy, which failed to resolve the psoriasis flare. Treatment with a combination of apremilast 30 mg twice daily, triamcinolone acetonide 0.1% ointment, and intralesional triamcinolone acetonide injections (2.5 mg/mL) resulted in symptomatic improvement. During a follow-up visit in January 2025, the patient reported an itch numeric rating scale of 0 and did not have any psoriatic lesions.

## Discussion

This case examines worsening of psoriasis in a patient with a recent streptococcal infection, undergoing IL-17 inhibitor treatment. The latency period of the psoriasis flare, occurring within a week of a streptococcal infection and four months since starting on the IL-17 inhibitor trial, adds a layer of complexity in treatment management as it supports either a post-streptococcal or IL-17 inhibitor-induced psoriasis flare [9,10]. Most cases of upper respiratory tract infection induced psoriasis flare manifest as an acute onset of guttate papules and self-resolve within weeks to three months, though it may persist beyond this timeframe [2,11]. Paradoxical psoriasis from biologic therapy is a rare occurrence, with the literature limited mostly to case reports and retrospective studies. Paradoxical psoriasis from IL-17 inhibitor use often shows palmoplantar involvement of plaque or pustular psoriasis, with female sex and concurrent autoimmune disease as general predisposing factors [9,12].

Due to limited data, no standardized treatment protocol exists for psoriasis flare-up from streptococcal infection or biologic therapy. For guttate psoriasis flares often triggered by a streptococcal infection, topical corticosteroids and calcipotriol cream in combination with phototherapy are well supported in the literature, with antibiotic treatment having minimal efficacy in the resolution of psoriasis [11]. When systemic therapy is indicated, methotrexate and cyclosporine continue to be the top choices, with the use of biologics being limited since most cases resolve within a short timeframe from the following treatments [11]. In our case, the patient’s psoriasis flare persisted for eight months and had failed to achieve the desired therapeutic benefit from potent steroids and phototherapy.

It is possible that continuation of the IL-17 inhibitor may have resolved her psoriasis; however, follow-up on paradoxical reactions shows mixed data. The case-based review by Chaitidis et al. indicated that paradoxical psoriasis showed satisfactory responses both in patients who continued IL-17 inhibitors with adjunctive therapy and in those who discontinued and initiated alternative systemic therapy [9]. However, another case-based review by Wang et al. recommended against reintroducing IL-17 inhibitors for paradoxical psoriasis due to a lack of clinical benefit and potential deterioration [8]. In the case-based reviews, the following systemic agents were reported: steroids, cyclosporine, acitretin, methotrexate, tofacitinib, upadacitinib, or another class of biologic agent [8,9]. Our case suggests apremilast may also be a useful alternative in persistent psoriasis flares in patients who fail to respond to biologic agents, aggressive steroids, phototherapy, tyrosine kinase 2 receptor inhibitors, or have contraindications against conventional systemic treatment with methotrexate or cyclosporine.

A proposed mechanism leading to paradoxical psoriasis is that the targeted blockade of IL-17 receptors causes cytokine rearrangements that favor inflammation and proliferation of keratinocytes [8]. Phosphodiesterase 4 (PDE4) inhibitors like apremilast may circumvent this issue since it causes broad level immunomodulation by increasing levels of cAMP in cells, which in turn reduces multiple pro-inflammatory cytokines and upregulates anti-inflammatory cytokines, particularly interleukin-10 (IL-10) [13]. Apremilast shows moderate clinical efficacy in the treatment of plaque psoriasis, with its results comparable to methotrexate for difficult-to-treat variants like palmoplantar psoriasis [6,14,15]. Apremilast has a good safety profile and does not typically require lab monitoring [16]. While comparative analysis indicates biologics have higher PASI 75, 90, 100 response rates than apremilast, the data often includes patients with classical plaque psoriasis [6,14,15]. Our case indicates further comparative data is needed to evaluate the management of complex cases, including patients with contraindications, flare-ups, guttate and palmoplantar type variants, and multiple failed treatments.

## Conclusions

Psoriasis is a chronic inflammatory condition often necessitating multiple modes of treatment for symptom management. In cases of psoriasis with an unknown origin of flare and multiple failed treatments, the use of apremilast may be a useful systemic treatment choice in combination with topical and intralesional corticosteroids. It is essential to adopt a personalized approach that minimizes the risk of adverse reactions while aiming for maximal therapeutic benefit.
